# Notes and News

**Published:** 1900-10-06

**Authors:** 


### NOTES AND NEWS.

(Collected and Communicated.)

A good number of medical men are standing for Parliament at this election, including Dr. Conan Doyle.

We are asked to announce that the British Dental Association is prepared, as usual, to receive applications for grants towards dental research. The applications should be sent to the Hon. Secretary, 40 Leicester Square, London, W.C.

The Bishop of Rochester has consented to preach the sermon at St. Paul's Cathedral on Wednesday, October 17, at 7.30 p.m., at the Annual Medical Service in State, organised by the Guild of St. Luke, and generally attended by many members of the medical profession. This is always an imposing ceremony by reason of the fact that many of the doctors appear in their academical robes. A choir of over 200 voices, provided by the London Gregorian Association, will render the music, so that the result this year, as in the past, is likely to be solemn, impressive, and brilliant. Admission to the space under the dome will be by ticket only.

At the last annual meeting of the Trustees of the Manchester Royal Infirmary, a committee was appointed to take eminent medical opinion as to the suitability of building the new Infirmary after other than the pavilion plan, with a view to economy of space. The committee accordingly addressed their inquiries to Lord Lister, Sir William Mac Cormac, Sir Thomas Smith, Sir Samuel Wilks, Professor McEwen, Professor Thomas Annandale, Professor Clifford Allbutt, Dr. Goodhart, Mr. Frederick Treves, Mr. Howard Marsh, and Mr. Timothy Holmes. Lord Lister, Sir Thomas Smith, Professor McEwen, and Sir William Mac Cormac voted in favour of the pavilion system above all others, but of the doctors who expressed their opinion, only two regarded such a system as really necessary to successful treatment and administration. The committee reported to the Board of the Hospital that it is their unanimous opinion that the adoption of the pavilion system is not necessary, and the Board have accepted their decision. The Board of Management have now received authority to secure plans and estimates for the erection of the Infirmary.

Two deaths from the plague during the last week are reported from Glasgow, leaving twenty-one patients under treatment in the hospital. Glasgow is still generally treated as suspect, as a port, and medical men from various parts are visiting the city to study the course of the disease and the measures and treatment adopted. Russia has sent a medical man and a bacteriologist, and very strict sanitary regulations are imposed upon any vessel visiting the port, in accordance with the various international conventions on this subject.

At the last week's meeting of the Edinburgh Royal Infirmary a letter was read from the Secretary of the F. G. Tait Memorial, stating that the General Committee did not approve of the proposal to endow a bed in the Edinburgh Infirmary as a memorial, seeing that no preference was promised to any class of patient selected. They preferred to devote the sum collected to the local hospital of St. Andrews, which would name a whole ward after Lieut. Tait.

It has given general satisfaction that the Government has definitely declared its intention that all permanently disabled soldiers are to have a pension of at least 1s. 6d. a day, so that no man robbed of the means of earning his living by serving his country in the army need seek the shelter of the workhouse. The assurance which Lord Lansdowne has given is a safeguard to the public as well as to the soldier, for henceforth they will know that harrowing tales of starvation from disabled warriors cannot be true. Further, while formerly pensions were frequently given for a limited period, in the future the permanently disabled will know from the first that they may count upon a certain provision for life.

The Royal London Ophthalmic Hospital is a severe sufferer from the heavy taxation which is levied upon medical charities. The institution at Moorfields is assessed at £870 in all. During the year since which the new hospital has been opened, the authorities have paid upwards of £500 to the rates, and being in difficulties respecting the payment of the £324 remaining, were eventually summoned to appear before the magistrate. Mr. Bland, the secretary, attended to plead the poverty of the hospital, where 138 beds are maintained and upwards of 40,000 patients are treated yearly. One of the overseers was present to explain that the hospital was not rated on a commercial basis, the charitable element being taken into account. Eventually the hospital authorities were ordered to pay within 14 days.

We have heard so much of the animosity of our foreign neighbours during our wars that it is especially desirable that deeds of friendliness from the same quarters should not be overlooked. It is not known to all how useful the French ambulance establishment was at Johannesburg. Although it was originally intended for the service of the Boers, it was not welcomed by them, and only came into full use after British occupation, when many of the fifty-six beds it contained were utilised by English officers and soldiers, who received the greatest kindness and attention. The English Tommies have also to thank several trade houses in Paris for many little comforts that reached them, as quite a substantial sum was contributed from this source to provide what was sent out through the *Daily Mail* Fund. The Italian Red Cross Society proved a most generous friend; through it sixty-six cases of necessaries and extras for the sick were sent to the Transvaal.

